# Repression of GW/P body components and the RNAi microprocessor impacts primary ciliogenesis in human astrocytes

**DOI:** 10.1186/1471-2121-12-37

**Published:** 2011-08-31

**Authors:** Joanna J Moser, Marvin J Fritzler, Jerome B Rattner

**Affiliations:** 1 Department of Biochemistry and Molecular Biology, Faculty of Medicine, University of Calgary, Calgary, Alberta, Canada; 2 Departments of Cell Biology; Anatomy and Oncology, Faculty of Medicine, University of Calgary, Calgary, Alberta, Canada

**Keywords:** centrosome, centriole, basal body, primary cilia, P-bodies, GW182, Ago2, Drosha, DGCR8, siRNA, miRNA

## Abstract

**Background:**

In most cells, the centriolar component of the centrosome can function as a basal body supporting the formation of a primary cilium, a non-motile sensory organelle that monitors information from the extracellular matrix and relays stimuli into the cell via associated signaling pathways. Defects in the formation and function of primary cilia underlie multiple human diseases and are hallmarks of malignancy. The RNA silencing pathway is involved in the post-transcriptional silencing of > 50% of mRNA that occurs within GW/P bodies. GW/P bodies are found throughout the cytoplasm and previously published live cell imaging data suggested that in a malignant cell type (U2OS), two GW/P bodies reside at the centrosome during interphase. This led us to investigate if a similar relationship exists in primary cells and if the inhibition of the miRNA pathway impairs primary cilium formation.

**Results:**

Two GW/P bodies as marked by GW182 and hAgo2 colocalized to the basal body of primary human astrocytes as well as human synoviocytes during interphase and specifically with the distal end of the basal body in the pericentriolar region. Since it is technically challenging to examine the two centrosomal GW/P bodies in isolation, we investigated the potential relationship between the global population of GW/P bodies and primary ciliogenesis. Astrocytes were transfected with siRNA directed to GW182 and hAgo2 and unlike control astrocytes, a primary cilium was no longer associated with the centrosome as detected in indirect immunofluorescence assays. Ultrastructural analysis of siRNA transfected astrocytes revealed that knock down of GW182, hAgo2, Drosha and DGCR8 mRNA did not affect the appearance of the earliest stage of ciliogenesis but did prevent the formation and elongation of the ciliary axoneme.

**Conclusions:**

This study confirms and extends a previously published report that GW/P bodies reside at the centrosome in U2OS cells and documents that GW/P bodies are resident at the centrosome in diverse non-malignant cells. Further, our study demonstrates that repression of key effector proteins in the post-transcriptional miRNA pathway impairs primary cilium formation.

## Background

In most eukaryotic cells the centrosome, composed of centrioles and associated pericentriolar material (PCM), acts as a major microtubule organizing center (MTOC) participating in the organization of both the interphase cytoskeleton and the mitotic spindle. In addition, the centriole component of the centrosome can function as a basal body that organizes the formation of a cilium while in many cases the associated PCM continues to operate as a cytoplasmic MTOC. This cilium can be one of two types, a motile cilium with a 9+2 arrangement of microtubules or non-motile (primary) cilia with 9+0 arrangement of microtubules (reviewed in [1]). Most vertebrate cells contain a single non-motile primary cilium that is assembled in a step-wise manner from the distal end of a mature centriole within the centrosome. We have previously shown that primary ciliogenesis in cultured human astrocytes and synoviocytes proceeds through stages beginning with the formation of a membrane bound vesicle at the distal end of the basal body (here referred to as stage 1) followed by the establishment and growth of a 9+0 ciliary axoneme [2,3]. Although long ignored, the primary cilium has recently been the focus of intense investigation. These efforts have established that the primary cilium is a key coordinator for a variety of signaling pathways that function in development and tissue homeostasis. Importantly, defects associated with the primary cilium underlie a variety of human diseases and developmental disorders including Alström, Bardet-Biedl, Joubert, Meckel-Gruber and Oral-facial-digital type 1 syndromes where common clinical phenotypes include obesity, ataxia and mental retardation, suggesting that primary cilia are required for the proper development and particularly function in the brain [4].

Proteins involved in cell cycle progression are also linked to primary cilium expression (for review see [5-7]) and may play a role in tumor formation as reported in two recent studies [8,9]. Further, defects in early stages of ciliogenesis were reported as a common feature in astrocytoma/glioblastoma cells including highly malignant T98G glioblastoma multiforme cells which expressed abnormally long centrioles and no primary cilia as evidenced by ultrastructure analysis [2]. To date, numerous cilia or basal body-associated proteins have been discovered by proteomic, comparative genomic and bioinformatic studies, representing the so-called "ciliome" [10,11]. A recent functional genomics screen has identified potential modulators of ciliogenesis in telomerase-immortalized human retinal pigmented epithelial cells, however the molecules and/or pathways that regulate ciliogenesis remain to be identified.

Centrioles/basal bodies and the primary cilium region are associated with a variety of cellular organelles (e.g. Golgi apparatus, nucleus) and endocytosis signaling pathways in a cell cycle specific manner, which frequently is dependent upon a functional interdependence between the centrosome/basal body and these organelles [2,3,12]. Recently, a real time microscopy study by Aizer *et al*. documented that a pair of stationary GW/P bodies reside at the centrosome in human U2OS (osteosarcoma) interphase cells, a feature that is markedly different from the majority of cytoplasmic GW/P bodies that are mobile and can transit rapidly along microtubule tracks to and from the centrosome [13].

GW/P bodies are cytoplasmic ribonucleoprotein granules that contain messenger RNA (mRNA), microRNA (miRNA) and proteins involved in mRNA transport, stabilization, silencing and/or degradation (reviewed in [14,15]). Key GW/P body constituent proteins, GW182 and hAgo2, are cytoplasmic effector proteins of the RNA silencing pathway that bind miRNA to silence and degrade mRNA [16-18]. The observation by Aizer *et al*. that two stationary GW/P bodies reside at the centrosome in human malignant cells [13] raises the possibility that miRNA could potentially be located at the centrosome in GW/P bodies and may play a role in posttranscriptional regulation of mRNA involved in centrosome functions including primary ciliogenesis. The idea that RNA may be present at the centrosome is not entirely new and is consistent with reports showing that RNA is a component of the centrosome in the mollusc *Ilyanassa obsoleta *and in the surf clam (*Spisula solidissima*) [19-21] which leads us to hypothesize that GW/P bodies and the miRNA pathway may play a yet-to-be-determined role in centrosome function such as the expression of a primary cilium. The goal of this study was to determine if stationary GW/P bodies are also present at the centrosome of non-malignant cells particularly those capable of expressing a primary cilium and to determine if the inhibition of the miRNA pathway impairs cilium formation.

## Results and Discussion

### Two GW/P bodies localize to the centrosome/basal body in cultured human astrocytes and synoviocytes

In a previous study we investigated the expression of primary cilia in both normal primary astrocytes and human synoviocytes [2]. These studies demonstrated that in both cell types ciliogenesis is characterized by a series of morphological stages including an early stage during which the basal body is associated with a vesicle at the distal end (Stage 1) and subsequent stages (Stages 2-5) that show various degrees of ciliary axoneme development [2]. Unlike kidney primary cilia where the mature axoneme extends completely free of the plasma membrane, the proximal portion of the primary cilium in these two cell types is contained within a membrane channel contiguous with the plasma membrane (termed the "cilium-pit") that is integrated with the centrosome/basal body region via microtubules, is associated with Golgi complexes and is a major site of endocytosis [2,3].

**Figure 1:**
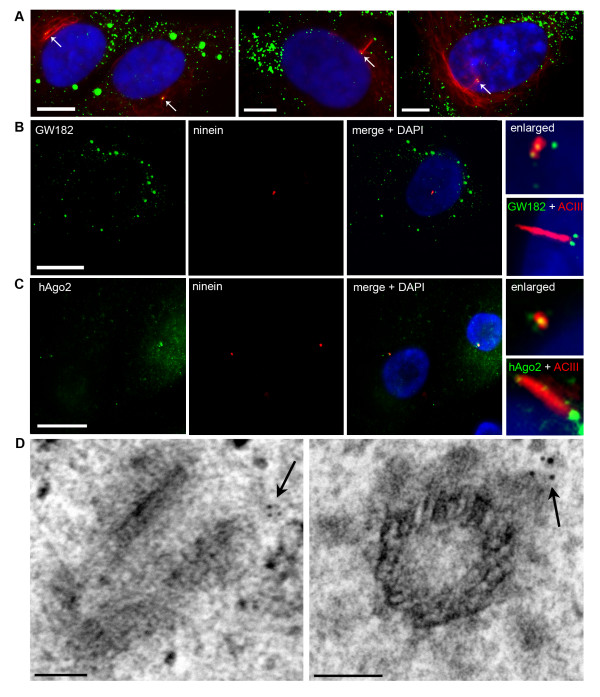
**RNA silencing proteins, GW182 and hAgo2, localize to the centrosome/basal body in human astrocytes throughout interphase**. (A) Astrocytes double labeled with mouse anti-acetylated tubulin (red) and human GW182 antiserum 18033 (green) and counterstained with DAPI (blue). Left panel illustrates two daughter cells. Cells were seeded at a low density to allow the identification of daughter cells following the completion of mitosis (early G1). Each cell contains a centrosome with associated GW/P bodies (arrows). Centre panel illustrates a cell in G1/S phase (the period of primary cilium expression). One of the two GW/P bodies is demonstrated in this focal plane at the basal body (arrow). Right panel illustrates a late S/G2 cell with prominent chromatin condensation (DAPI) and a centrosome with two GW/P bodies (arrow). (B) GW182 and (C) hAgo2 localization to the centrosome and the basal body of primary cilia was examined by IIF using 18033 and mouse monoclonal 4F9 to recombinant hAgo2 [29] (green). Nuclei were marked by DAPI. The inset box in (B) shows the location of GW182 marked by mouse monoclonal 4B6 antibody and (C) hAgo2 mouse monoclonal antibody relative to the primary cilia. All IIF scale bars = 15 μm. (D) GW182 localizes to the pericentriolar region in immunoelectron micrographs. Arrows indicate the immunogold stained GW/P body foci. EM scale bars = 100 nm.

To confirm and extend the report by Aizer *et al*. [13] we examined the position of GW/P bodies relative to the centrosome in interphase primary human astrocytes and synoviocytes. Since results were identical for both astrocytes and synoviocytes, here we only report the data obtained from the primary human astrocytes. Indirect immunofluorescence (IIF) analysis in over 100 interphase cells showed that, in addition to abundant GW/P bodies in the cytoplasm, GW/P body foci localize to the centrosome in 100% of cells that were maintained in a log phase culture, a frequency that is consistent with GW/P bodies being components of the centrosome [13]. Figure 1A illustrates the colocalization of GW/P bodies with the centrosome in daughter cells in early G1 phase, a late G1/S phase cell expressing a primary cilium and a late S/G2 cell displaying prominent chromatin condensation. Figure 1B shows in greater detail two GW/P bodies positioned at the centrosome and at the basal body during the expression of a primary cilium. These two foci were also highlighted by mouse monoclonal antibodies to the GW/P body component hAgo2 (Figure 1C and enlarged inset). Interestingly, hAgo2 also localized to the proximal portion of the shaft of the primary cilium (Figure 1C and inset). These IIF experiments were replicated using anti-glutamylated (glu) tubulin as an alternate marker of primary cilia (data not shown). It is important to note that a large number of GW/P bodies (up to ~50 per cell) are characteristic of primary human astrocytes (Moser JJ, unpublished data). The number of GW/P bodies observed in these cells differs greatly from those in malignant cells (astrocytoma/glioblastoma cell line, HeLa, HEK, U2OS) and seemingly correlates with their extensive cytoplasmic area. Further, the number of GW/P bodies elicited by IIF staining did not differ when GW/P bodies are over-expressed using GW182 GFP-cDNA (Moser JJ, unpublished data).

Immunogold electron microscopy confirmed that GW/P body foci, as marked by prototype GW182 human antiserum 18033, localized to the centrosome and further clarified that they are commonly found at the distal end of the centriole in the pericentriolar region as shown in longitudinal and transverse sections (Figure 1D, arrows). Taken together with previously published reports [13], these data suggest that two GW/P bodies are constitutive components of the centrosome during interphase including periods of primary cilium expression.

### Repression of GW182, hAgo2, Drosha and DGCR8 with siRNA inhibits the formation of mature primary cilia in human astrocytes

**Figure 2:**
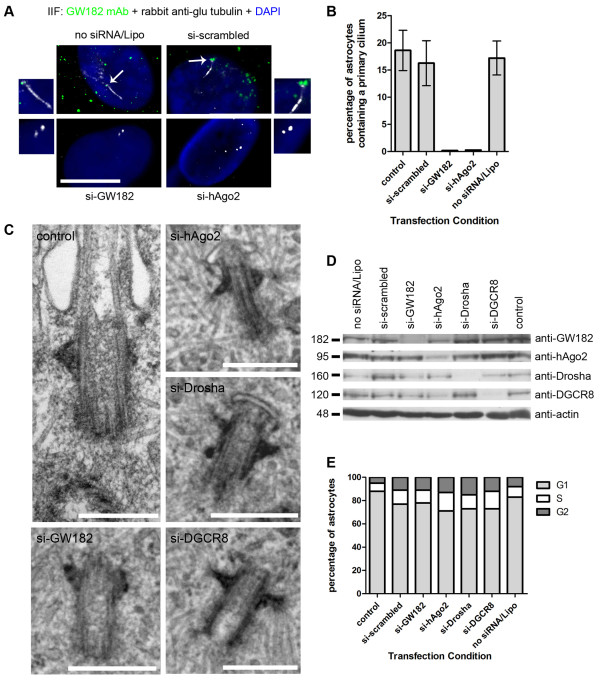
**SiRNA knockdown of RNAi components, GW182, hAgo2, Drosha and DGCR8, inhibits ciliogenesis in human astrocytes**. (A) The primary cilium was examined by IIF using antibodies to glu tubulin (white) in astrocytes transfected with no siRNA/Lipofectamine, scrambled siRNA and si-GW182 and double stained for GW bodies using mouse monoclonal 4B6 antibody to GW182 (green). The primary cilium as marked by glu-tubulin (white) was also examined in astrocytes transfected with si-hAgo2 and double stained with mouse monoclonal 4F9 antibody to hAgo2 (green). Nuclei were stained by DAPI. The merge + DAPI images were enlarged to focus on the cilium and centrioles relative to GW182 and hAgo2 staining. Scale bar = 15 μm. (B) The percentage of astrocytes containing primary cilia were quantitated from 5 independent IIF experiments containing > 250 cells described in (A). Bars represent the standard deviation of the mean. (C) Ultrastructural analysis of control astrocytes, scale bar = 400 nm, si-GW182, si-hAgo2, si-Drosha and si-DGCR8 treated astrocytes scale bars = 500 nm. (D) Human astrocytes were transfected for 30 hours with 100 nM siRNA and Lipofectamine 2000 (Invitrogen), lysed and processed for western blot analysis. The cells in the no siRNA/Lipofectamine lane were treated with Lipofectamine 2000 only (no siRNA) and the control lane represented untreated cells. (E) Cell cycle analysis showing the percentage of human astrocyte cells in G1, S and G2 phase during normal growth conditions (control) and under 100 nM siRNA transfected conditions.

We next investigated the effect of the inhibition of the miRNA pathway on cilia formation targeting the general GW/P body population since it is technically challenging to specifically target the two centrosomal GW/P bodies. Human astrocytes were transfected with 100 nM of siRNA directed to GW182 and hAgo2 as well as scrambled non-targeting siRNA and Lipofectamine only (no siRNA) for 30 and 70 hours. Non-treated control and siRNA-treated astrocytes were fixed and examined by IIF for primary cilia expression using antibodies directed against glu tubulin (Figure 2A). The specificity of the knockdown was monitored using mouse monoclonal 4B6 to GW182 (for si-GW182 conditions only) and mouse monoclonal 4F9 to hAgo2 (for si-hAgo2 conditions only) (Figure 2A). In the no siRNA/Lipofectamine only and si-scrambled treatment conditions, mature primary cilia were observed (white) with two GW/P body foci as marked by GW182 (green) localized to the basal body (arrows) (Figure 2A). After treatment with GW182 siRNA, two centrioles per cell were still visible by conventional microscopy as marked by anti-glu tubulin, however primary cilia were completely absent (Figure 2A). Similarly, primary cilia were absent and only two centrioles per cell were visible as marked by anti-glu tubulin when astrocytes were treated with si-hAgo2 (Figure 2A). The si-GW182 and si-hAgo2 transfected cells knocked down the expression of GW/P bodies and no green foci were present when stained using GW182 or hAgo2 mouse monoclonal antibodies by IIF (Figure 2A). These experiments were performed in triplicate and > 250 cells were counted for primary cilia expression under no treatment control, scrambled, no siRNA/Lipofectamine only, si-GW182 and si-hAgo2 conditions (Figure 2B). Quantitation of primary cilia examined by IIF showed that ~18% of control astrocytes displayed a mature cilium and under si-scrambled and no siRNA/Lipofectamine only conditions, 16% and 17% of cells displayed a mature cilium respectively (Figure 2B). By comparison, 0% of the cells displayed a mature cilium when treated with siRNA to repress GW182 or hAgo2 expression at 30 hours (Figure 2B) and 70 hours (data not shown) post transfection. Our results show that when GW/P bodies, including centrosome/basal body GW/P bodies, were repressed by cognate siRNA to GW182 and hAgo2, primary cilium formation was impacted.

To further examine the effect of our experimental manipulation, an electron microscopic (EM) study was undertaken, which showed that control astrocytes had a centrosome/basal body -primary cilium with a morphology similar to that previously reported (compare Figure 2C with the results reported in Figure 3 from [2]). However, after repression of GW182 and hAgo2 by cognate siRNA, cells expressing a mature primary cilium were no longer detected although basal bodies displaying a morphology characteristic with the earliest stage of ciliogenesis (Stage 1) were observed (Figure 2C, siGW182 and si-hAgo2 panels) indicating that the inhibition of the miRNA pathway impairs primary ciliogenesis at a specific point in its morphogenesis.

The truncation of ciliogenesis following the disruption of GW/P body components led us to question whether the expression of upstream nuclear components of the RNA silencing pathway such as the microprocessor proteins Drosha and DGCR8 would also affect ciliogenesis and thus support our initial observations. Both light and electron microscopic analysis of primary astrocytes treated with Drosha and DGCR8 siRNA showed similar phenotypes to that observed with GW182 and hAgo2 knockdown experiments (Figure 2C, light microscopy data not shown). Western blot analysis confirmed that the expression of GW182, hAgo2, Drosha and DGCR8 proteins were repressed after treatment with 100 nM of their respective cognate siRNAs for 30 hours (Figure 2D). The hAgo2 protein levels were decreased by ~60-70% after treatment with si-hAgo2 whereas GW182, Drosha and DGCR8 protein levels were decreased by ~85-95% after treatment with their cognate siRNAs (Figure 2D). Western blot analysis of the scrambled non-targeting siRNA/Lipofectamine only and no treatment control lysates showed that the siRNA repressive effects were specific (Figure 2D). Western blotting with anti-actin demonstrated that equal amounts of protein (40 μg) were loaded in each treatment condition (Figure 2D). The western blot analysis was repeated after 70 hours transfection with results identical to 30 hours transfection (data not shown). Taken together, the western blot results show that the cognate siRNAs for GW182, hAgo2, Drosha and DGCR8 knocked down the expression of their intended target with considerable efficiency and had no observable off-target repressive effects. The western blot data confirms our IIF results.

Finally, since ciliogenesis is tightly correlated with cell cycle progression and primary cilia are expressed predominantly during late G1/S, we performed a series of experiments to investigate the possibility that siRNA transfection was disrupting the cell cycle and hence ciliogenesis. Primary human astrocytes under control and siRNA transfection conditions were fixed and examined by flow cytometry following the addition of propidium iodide (Figure 2E). Astrocytes with no siRNA treatment and no siRNA/Lipofectamine had 88% and 83% of the cells in the G1 phase of the cell cycle, respectively (Figure 2E). Similarly, astrocytes treated with scrambled non-targeting siRNA had 77% of cells in G1 phase (Figure 2E). Astrocytes treated with siRNAs to GW182, hAgo2, Drosha and DGCR8 had 78%, 71%, 73% and 73% of the cells in the G1 phase respectively (Figure 2E), which was similar to results obtained from control samples. The results at 30 hours transfection were the same after 70 hours transfection (data not shown). These observations indicate that siRNA transfection does not change the cell cycle profile of human astrocytes to an extent that would elicit a decrease in primary cilia expression or impact the interpretation of our transfection data.

One drawback of our study is that current techniques do not allow us to specifically target the two GW/P bodies that reside at the centrosome and targeting the general GW/P body population with its possible secondary effects makes it difficult to draw a clear direct functional link between centrosomal GW/P bodies and centrosome functions such as cilium formation. We are currently attempting to address this issue using alternative experimental approaches. We examined GW/P body specific miRNA normalized to total cellular miRNA in human astrocytes and found that ten different miRNAs were differentially expressed in GW/P bodies as compared to the total cellular miRNA fraction and are predicted using Ingenuity Pathway Analysis software (IPA) to regulate mRNA involved in primary ciliogenesis. Six miRNA RNA-immunoprecipitated in GW/P bodies were predicted to regulate KIF3A (kinesin family member 3A) and include miR-29, miR-199, miR-181, miR-455, miR-155 and miR-145. Three miRNA RNA-immunoprecipitated in GW/P bodies were predicted to regulate VHL (von Hippel-Lindau tumor suppressor) and include miR-320, miR-368 and miR-143 and one miRNA RNA-immunoprecipitated in GW/P bodies was predicted to regulate ODF2 (outer dense fiber of sperm tails 2), KIF3Aand VHL and include miR-154. All three of these mRNA are involved in the formation and assembly of primary cilia [22-27]. Our preliminary results indicate that GW/P bodies contain miRNA that could potentially regulate mRNA critical for primary ciliogenesis (Moser JJ and Fritzler MJ, unpublished observations) thus raising the possibility that centrosome GW/P bodies are required for centrosome/basal body functions, opening a new area of investigation.

## Conclusions

The real time imaging results of Aizer *et al*. [13] indicated that a population of GW/P bodies were resident at the centrosome of U2OS cells. This observation raised the question as to whether this relationship is also found in other cell types and if so if the inhibition of the miRNA pathway impairs centrosome based functions. Our study confirms and extends the observation of Aizer and co-workers indicating that there is a population of centrosome based GW/P bodies in diverse non-malignant cell types. Further, we document that the inhibition of the miRNA pathway impairs ciliogenesis halting it at a stage prior to the initiation of axoneme formation.

## Methods

### Cells

Primary human cerebral cortex astrocyte cells (ScienCell Research Laboratories, Carlsbad, CA) were cultured in cell specific astrocyte medium containing 2% fetal bovine serum, 1% astrocyte growth supplement and 1% penicillin/streptomycin (ScienCell Research Laboratories). Cells were used between passages 4 - 6. Synoviocytes were cultured as previously described [3].

### Indirect Immunofluorescence (IIF)

Cells were cultured on poly-L-lysine coated coverslips (BD Falcon) for approximately 24 hours at 37°C and then fixed in 100% ice cold methanol for 10 minutes. Cells were blocked in 10% normal goat serum (NGS; Antibodies Incorporated, Davis, CA) and 2% bovine serum albumin (BSA; Sigma-Aldrich) for 30 minutes at room temperature (RT) and incubated with primary antibodies at appropriate working dilutions for 1 hour at RT.

Primary cilia were marked by three antibodies: rabbit anti-adenylyl cyclase III (ACIII) antibody (Santa Cruz Biotechnology, Inc., Santa Cruz, CA), rabbit anti-glutamylated (glu) tubulin (Chemicon, Temecula, CA) and mouse anti-acetylated tubulin (Sigma, St. Louis, MO). Human prototype GW182 antiserum 18033 was obtained from the Mitogen Advanced Diagnostics Laboratory (University of Calgary, Calgary, AB, Canada). Mouse monoclonal antibody 4B6 to recombinant GW182 and 4F9 to recombinant hAgo2 were obtained and used as previously described [28,29].

After washing with phosphate buffered saline (PBS), cells were incubated for 1 hour in a dark chamber with the corresponding secondary goat fluorochrome-conjugated antibodies. Alexa Fluor (AF) 488 (green) or 568 (red) secondary antibodies were from Invitrogen. Slides were washed in several changes of PBS, cell nuclei counterstained with 4',6-diamidino-2-phenylindole (DAPI), mounted in Vectashield (Vector Laboratories, Burlingame, CA) and examined for IIF using a 100× objective on a Leica DMRE microscope equipped with epifluorescence and an Optronics camera. Appropriate IIF controls with no primary antibody or only one primary antibody or both secondary antibodies revealed no detectable bleed-through between microscope filter sets.

### siRNA Transfection

Lipofectamine 2000 (Invitrogen) was used for transient siRNA (Dharmacon RNAi Technologies, Lafayette, CO) transfection following the manufacturer's protocol. Astrocytes were grown to ~60% confluence and transfected with 100 nM siRNA. The siRNAs included: si-scrambled (siGENOME non-targeting siRNA pool #1, D-001206-13-20), si-GW182 (TNRC6A siGENOME siRNA, D-014107-01), si-hAgo2 (EIF2C2 ON-TARGETplus SMARTpool, L-004639-00-0005), si-Drosha (RNASEN ON-TARGETplus SMARTpool, L-016996-00-0005), si-DGCR8 (DGCR8 ON-TARGETplus SMARTpool, L-015713-00-0005). Cells were fixed or lysed at 30 hours and 70 hours after transfection.

### Western Blot

Primary human astrocytes were cultured as outlined above, harvested, pooled, and pelleted by centrifugation at 196 *g *for 5 minutes and washed several times with ice-cold sterile PBS. The cell pellets were resuspended, mixed in an equal pellet volume of PBS containing Complete EDTA-Free Protease Inhibitor (Roche, Mannheim, Germany), and then lysed by sonication in a water bath for two 80 second bursts. Finally, the viscous lysates were sheared by passing them through a syringe fitted with a 21G 1.5-inch needle and centrifuged in a tabletop microfuge at 16,000 *g *for 10 minutes at 4°C. The supernatant was collected and stored at -20°C or -70°C for short term or long term use, respectively. The amount of protein in each sample was quantitated using a NanoVue spectrophotometer (GE Healthcare Life Sciences, Baie d'Urfe, QC) and 40 μg of protein was resolved in a 10% sodium dodecyl sulphate - polyacrylamide gel, electrophoretically transferred to nitrocellulose membranes (Bio-Rad Laboratories, Hercules, CA) and then processed for western blot analysis. Nitrocellulose membranes were blocked for 1 hour in 5% DuraUse anti-spoil skim milk (SignaGen Laboratories, Gaithersburg, MD) in PBS-T (0.05% Tween-20) and overlayed with the primary antibody at the appropriate dilution for 1 hour at RT. Primary antibodies for western blot analysis were monoclonal antibody 4B6 to recombinant GW182 [28], monoclonal antibody 4F9 to recombinant hAgo2 [29], goat antibodies to Drosha and DGCR8 (Santa Cruz Biotechnology, Inc.) and rabbit antibody to actin (Sigma). Following washing with PBS-T for a total of 30 minutes, membranes were overlayed with secondary horseradish peroxidase (HRP)-conjugated antibodies, either goat anti-human (diluted 1:20,000; Sigma) or goat anti-rabbit (diluted 1:20,000; Jackson ImmunoResearch, West Grove, PA) for 1 hour at RT. Membranes were washed with PBS-T and subsequent identification of bound antibody was detected using the Amersham enhanced chemiluminescence (ECL) kit (GE Healthcare). Reactive proteins were visualized by exposure and recording on Amersham Hyperfilm ECL scientific imaging film (GE Healthcare).

### Cell Cycle Analysis

Astrocytes (1 × 10^6^) were washed in PBS, resuspended in 500 μl PBS, fixed in 500 μl of 100% ethanol for 24 hours at 4°C, centrifuged at 200 *g*, washed in PBS and resuspended in 500 μl propidium iodide staining buffer (50 μg/ml propidium iodide, 0.1% Triton X-100, 0.2 mg DNase free RNase A in PBS) for 45 minutes at RT prior to flow cytometry analysis at the University of Calgary Flow Cytometry Lab (University of Calgary, Calgary, AB).

### Immunogold Electron Microscopy

Cells were seeded into 35 mm dishes grown to confluence as monolayers over two days, then washed in PBS and fixed in 3% paraformaldehyde and 3% glutaraldehyde in Millonig's phosphate buffer for 1 hour at RT. Post-fixation was in 2% OsO_4 _for 20 minutes at RT. The ultrathin imbedded sections were placed directly onto carbon-coated 200-mesh nickel grids. Each grid was hydrated with distilled H_2_O and processed by incubating with fresh saturated solution of 0.22 μm-filtered sodium metaperiodate for 15 minutes at RT. After washing with distilled H_2_O, sections were incubated with 0.1 N HCl for 10 minutes at RT. After washing with PBS, sections were blocked in 0.1% BSA and 5% NGS in PBS for 1 hour at RT and incubated with primary antibody 18033 diluted 1:1000 in 0.1% BSA and 1% NGS in PBS overnight at 4°C. Sections were washed 3 times with 0.1% BSA and 1% NGS in PBS and incubated with secondary 10 nm gold anti-human IgG (BB International, UK) diluted 1:100 in 0.1% BSA/PBS for 45 minutes at RT. Washing was repeated 3 times with 0.1% BSA and 1% NGS in PBS, once with PBS alone for 5 minutes at RT and with distilled H_2_O an additional 5 times. Sections were contrasted with uranyl acetate and lead citrate and then examined in a H-700 Hitachi electron microscope. Controls included incubation with secondary antibody alone and human serum antibodies to the centromere (data not shown).

### Electron Microscopy

Astrocytes were seeded into 35 mm dishes grown to confluence as monolayers over two days, then washed in PBS and fixed in 3% glutaraldehyde in Millonig's phosphate buffer for 1 hour at RT. Post-fixation was in 2% OsO_4 _for 20 minutes. The cells were dehydrated in ethanol, and then infiltrated with Polybed 812 resin (Polysciences Inc., Warrington, PA). Polymerization was performed at 37°C for 24 hours. Silver-gray sections were cut with an ultramicrotome (Leica) equipped with a diamond knife, stained with uranyl acetate and lead citrate and then examined in a H-700 Hitachi electron microscope.

## Authors' contributions

JJM carried out cell culturing for all experiments, performed the indirect immunofluorescence, siRNA transfection, western blot and flow cytometry assays and wrote the first draft of the manuscript. JBR carried out the electron microscopy studies and performed indirect immunofluorescence experiments. All authors conceived the study, participated in the design and interpretation of the data, read, edited and approved the final manuscript.
